# Plant-based dietary patterns, micronutrient status and breast cancer outcomes: a joint analysis of UK Biobank and Chinese longitudinal healthy longevity survey

**DOI:** 10.3389/fnut.2025.1748611

**Published:** 2026-01-26

**Authors:** Weizhe Xu, Wen Gu, Yanqiu Huang, Shuli Li, Honglin Liu, Xun Zhu

**Affiliations:** 1 Department of Breast and Thyroid Surgery, The Second Affiliated Hospital of Soochow University, Suzhou, China; 2 Department of Clinical Laboratory, Zhongshan Second People's Hospital, Zhongshan, China; 3 Department of Infectious Diseases and Public Health City University of Hong Kong, Hong Kong, Hong Kong SAR, China

**Keywords:** breast cancer, CLHLS, machine learning, micronutrients, mortality, plant-based diets, UK Biobank

## Abstract

**Background:**

Plant-based diets may lower breast cancer risk, but their impact on breast cancer-related mortality is unclear. We explored associations of plant-based dietary patterns (Healthful Plant-Based Diet Index [HPDI/PDI]) and micronutrient intake with breast cancer incidence and all-cause mortality in patients.

**Methods:**

Using data of UK Biobank (UKB; 67,045 cancer-free participants; 3,397 breast cancer patients) and Chinese Longitudinal Healthy Longevity Survey (CLHLS), we analyzed dietary scores and micronutrient intake via multivariate Cox regression, restricted cubic splines, and predictive models (concordance index, Random Forest, and time-dependent ROC).

**Results:**

Among 67,045 breast cancer-free participants, the highest HPDI tertile was associated with 11% lower breast cancer risk (HR = 0.89, 95%CI: 0.82–0.98) vs. lowest tertile (4% reduction per SD increase, HR = 0.96, 95%CI: 0.93–1.00). Among 3,397 breast cancer patients, the highest HPDI tertile showed 28% lower mortality (HR = 0.72, 95%CI: 0.55–0.95) vs. lowest (11% reduction per SD, HR = 0.89, 95%CI: 0.79–1.00). Individuals with high PDI scores exhibited a 39% lower risk of cancer compared to those with low scores in CLHLS (HR = 0.61, 95%CI: 0.41–0.92). Higher intakes of vitamins B2 and C, calcium, and magnesium were inversely associated with risk and mortality, while each SD increase in sodium raised mortality risk by 15% (HR = 1.15, 95%CI: 1.01–1.32). Predictive models showed optimal 5-year performance overall; micronutrients alone best predicted breast cancer risk across timepoints, while HPDI peaked for 5-year mortality prediction (AUC = 0.625). The combined model achieved superior 10-year prognosis.

**Conclusions:**

High adherence to a healthful plant-based diet, together with sufficient intake of key micronutrients and reduced sodium consumption, may contribute to breast cancer prevention and improved survival outcomes.

## 1 Introduction

Breast cancer remains one of the most common malignancies globally and a leading cause of cancer-related death among women. Despite advances in detection and treatment, its persistent incidence and mortalitypose a significant challenge to public health worldwide (1). Consequently, the exploration and identification of modifiable lifestyle factors, particularly dietary patterns, are crucial for both the primary prevention and secondary prognosis of breast cancer.

In recent years, Plant-Based Dietary Patterns have garnered increasing attention. These eating styles, such as the Healthful Plant-Based Diet Index (HPDI) and the Alternate Mediterranean Diet (AMED), emphasize higher consumption of fruits, vegetables, whole grains, and legumes, while limiting red and processed meats. These diets are believed to exert anti-inflammatory and anti-carcinogenic potential due to their richness in antioxidants and bioactive compounds. Numerous prospective cohort studies and meta-analyses have confirmed an inverse association between higher adherence to healthful plant-based diets and the risk of developing various cancers, including breast cancer (2, 3). Notably, a Chinese cohort study has reported a potential protective role of calcium intake against breast cancer, likely mediated by calcium's inherent antiproliferative effects on cancer cells (4, 5). Huang et al. (20) found that increased magnesium intake may reduce breast cancer risk through downregulating C-reactive protein, an established marker of systemic inflammation linked to cancer progression (6). However, evidence gaps remain. First, most research has focused on breast cancer incidence, while studies on the impact of plant-based dietary patterns on all-cause mortality in patients already diagnosed with breast cancer are limited and often inconsistent. Second, prior work largely evaluates the holistic effect of dietary patterns, lacking a systematic and comprehensive assessment of the independent contribution of individual key micronutrients (e.g., specific vitamins and minerals) to breast cancer risk and patient prognosis. Third, the predictive capacity of dietary factors for long-term outcomes remains underexplored; conventional regression models may not capture complex non-linear relationships or variable interactions that modern machine learning approaches can address (7).

Using data from two large prospective cohorts of UK Biobank (UKB) and Chinese Longitudinal Healthy Longevity Survey (CLHLS), we aimed to: (1) evaluate the associations of HPDI and AMED with breast cancer incidence and all-cause mortality in the UK and China; (2) examine the independent associations of key micronutrient intake (including specific vitamins and minerals) with these outcomes; (3) compare the long-term predictive performance of dietary patterns and key micronutrients, individually and jointly, utilizing multidimensional statistical approaches including Cox regression, Restricted Cubic Splines (RCS), and multiple machine learning models (such as Random Forest and time-dependent ROC analysis). These findings may inform precision nutrition strategies for breast cancer prevention and survivorship in the UK and China.

## 2 Methods

### 2.1 Study population and design

This study utilized data from the UKB, a large-scale biomedical resource encompassing the entire United Kingdom. The UKB collected genetic data, lifestyle information, biological samples, and health records from 500,000 participants. After excluding individuals who were male, had incomplete dietary information, missing outcome indicators, or incomplete covariate data, the analytical cohort comprised 67,045 participants without breast cancer and 3,397 patients with breast cancer (Supplementary Figure S1). All participants provided written informed consent. CLHLS is a community-based prospective cohort study conducted among elderly people in China. It collected various data related to population characteristics, lifestyle, health outcomes, and more. This study used food frequency information data measured in the 2008 wave as the baseline and followed up on disease outcomes in the 2011, 2014, and 2018 waves. Participants who meet the following criteria were included in the analysis: (1) complete data of cancer definition and without cancer in 2008, (2) complete data from simplified FFQ in 2008, (3) completion of at least one follow-up after the 2008 wave, and (4) complete data of covariates. Finally, a total of 7,431 eligible participants were included in the analysis, of which 114 participants developed cancer during follow-up (Supplementary Figure S2).

### 2.2 Definition of plant-based diets and micronutrients

Diet was assessed using the validated web-based Oxford WebQ, a 24-h dietary recall tool. A UKB sub-cohort completed this assessment on ≥1 of five occasions between April 2009 and June 2012. The Oxford WebQ has been validated against interviewer-administered 24-h recalls, yielding a mean Spearman correlation coefficient of 0.62 (range: 0.54–0.69) for macronutrients (8, 9). CLHLS used a simplified FFQ of 22 food group items to collect participants' dietary intake information. In the current study, a total of 16 food groups were used to evaluate plant-based dietary patterns.

Food consumption amounts were calculated by multiplying the reported quantity consumed by its assigned portion size. Nutrient intakes were derived by multiplying each food's consumption quantity by its nutrient content per portion (using McCance and Widdowson's The Composition of Foods and Supplements), then aggregating values across all food groups. For participants with multiple assessments, usual intake was estimated using average food and nutrient values.

#### 2.2.1 AMED score

This study assessed adherence to the Mediterranean diet using the AMED score, which quantifies intake across nine key components based on sex-specific median cutoffs. Beneficial components (e.g., vegetables, fruits, whole grains) scored 1 point for intake above the sex-specific median and 0 for below. Conversely, potentially harmful components (e.g., monounsaturated-to-saturated fatty acid ratio, red/processed meats, poultry) used reverse scoring (below median = 1; above = 0). Total scores ranged from 0 to 9, with higher values indicating stronger adherence to Mediterranean diet principles (10).

#### 2.2.2 HPDI

The HPDI score was calculated from 17 food groups (excluding vegetable oil, unavailable in UKB), with each group scored 1–5 based on intake quintiles. For plant-based foods (whole grains, fruits, vegetables, nuts, legumes, and tea/coffee), the highest quintile scored 5 and the lowest 1. Conversely, animal-based foods (animal fat, dairy, eggs, fish/seafood, meat, and other animal foods) followed reverse scoring (highest quintile = 1; lowest = 5). Other groups (refined grains, potatoes, sugary drinks, fruit juices, and sweets/desserts) scored 1 for the highest quintile. Total scores ranged from 17 to 85, with higher values indicating a healthier diet (11). In CLHLS, we used 16 food groups. For fruits and fresh vegetables, the intake frequency of “almost every day,” “quite often,” “occasionally,” or “rarely or never” corresponds to 5, 4, 2, and 1 points, respectively. For whole grains, refined grains, vegetable oils, and animal fats, the answer is recorded as a binary method (“whether as a staple food” and “whether as the main cooking oil”), corresponding to 5 or 1 point. Positive scores indicate that the higher the score, the higher the frequency of consumption. The plant-based food group of PDI received positive scores, while the animal based food group received reverse scores. Compared to PDI, HPDI received reverse scores for the unhealthy plant-based food group (refined grains, pickled vegetables, and sugar) (12). Total scores ranged from 16 to 80. In our analysis, we consider these two indices as categorical variables (measured in minimum 40% and maximum 60% of the population).

### 2.3 Definition of breast cancer and mortality

Cancer diagnoses were captured through linkage to national cancer and death registries. All outcomes were defined according to the World Health Organization's International Statistical Classification of Diseases (ICD-10), with breast cancer including C50 and D05. Person-years of follow-up were calculated from baseline assessment at recruitment to first registration of cancer, death, loss, or end of follow-up, whichever came first. Patients with breast cancer at baseline were either diagnosed with breast cancer prior to enrollment or self-reported. Person-years of follow-up for mortality outcome were calculated from baseline assessment at recruitment to death, loss, or end of follow-up, whichever came first (13). In CLHLS, this study used self-reported data from participants to obtain follow-up results for cancer.

### 2.4 Covariates

The study adjusted for the following covariates in the UKB cohorts: age (continuous), race (White, Asian/Asian British, Black/Black British, Chinese, Mixed, and Other), educational level (less than high school/high school or above), body mass index (BMI, continuous), total energy intake (continuous), Townsend deprivation index (tertiles:T1–T3), and lifestyle factors (smoking status: yes/no; drinking frequency: unknown/never/ < 1 time/week/1–7 times/week). For CLHLS, this study adjusted age (< 90, ≥90), gender (male, female), province (23 provinces and cities including Beijing, Tianjing, Hebei, and others), occupation (10 occupational statuses including professional and technical personnel and others), financial support (10 financial supports including retirement wages and others), educational level (classified by years of schooling: < 6, 7–8, 9–11, ≥12), drinking status (yes, no), physical activity (yes, no), history of CVD (yes, no), and SBP (continuous).

### 2.5 Statistical analysis

In the baseline characteristics, continuous variables were presented as mean ± standard deviation (SD), while categorical variables were described using counts (percentages). Group comparisons were performed using *t*-tests for continuous variables and χ^2^ tests for categorical variables. The study employed multidimensional statistical models to examine the associations between dietary factors and outcomes. Cox proportional hazards models were constructed with the first tertile of each dietary indicator as the reference. Two adjusted models were implemented: Model 1 adjusted for age, race and total energy intake; Model 2 additionally adjusted for BMI, Townsend deprivation index, education level, smoking status, drinking frequency. Results were reported as hazard ratios (HRs) with 95% confidence intervals (CIs). This study employed the RCS model to thoroughly investigate the dose-response relationship between dietary patterns and breast cancer and all-cause mortality. As a flexible non-parametric regression method, RCS excel in analyzing non-linear dose-response relationship between continuous exposure variables and outcomes, while effectively avoiding overfitting and multicollinearity (14). In this study, we used three knots at the 10th, 50th, and 90th percentile of the dietary patterns, with median dietary scores selected as reference values (15). To assess the statistical significance of non-linear association, likelihood ratio tests (LRT) were performed to calculate both non-linear *P* values and overall *P* values. RCS analyses were performed using the rms package in R. We compared the full RCS model (allowing non-linearity) with a linear nested model via likelihood ratio test (LRT), and the non-linear *P* value was derived from the LRT output of the anova() function in the rms package. A non-linear *P* value less than 0.05 was considered indicative of the non-linear relationship between exposure and outcome.

Based on the results from the Cox proportional hazards model, we focused on evaluating the predictive value of the HPDI and micronutrients (including Vitamin C, Calcium, Magnesium, and Copper for breast cancer; Vitamin B2, Calcium, Magnesium, Phosphorus, and Sodium for all-cause mortality). We constructed three distinct models: (1) the dietary model including HPDI; (2) the micronutrient model including micronutrients above; and (3) the combined model incorporating both HPDI and micronutrients. Similarly, to minimize potential confounding effects, all models were adjusted for age, ethnicity/race, total energy intake, Townsend deprivation index, drinking frequency, and educational level. The C-index of the corresponding Cox proportional hazards model were calculated to evaluate model fit (16). Additionally, we employed the Random Forest to predict the outcomes. Specifically, the dataset was randomly divided into training (70%) and testing (30%) sets at a 7:3 ratio, with 500 decision trees constructed. The predictive performance of the models was assessed by calculating the Area Under the ROC Curve (AUC). Finally, to evaluate the predictive capacity of the HPDI and micronutrients at different time points (3, 5, and 10 years) for breast cancer and all-cause mortality, time-dependent ROC analysis was conducted (17). ROC curves were plotted to visually compare the predictive efficacy across different models. Higher C-index and AUC values indicate superior predictive performance of the models.

All statistical analyses were conducted using R statistical software (version 4.5.0). The R packages utilized in the analyses included “plotRCS,” “randomForest,” “ggplot2,” “survival” and “pROC.” A two-sided *P* value < 0.05 was defined as statistically significant.

## 3 Results

### 3.1 Baseline characteristics of participants

Our analysis for UKB included 67,045 participants without breast cancer and 3,397 patients with breast cancer at baseline (Table 1). Compared to participants without breast cancer, patients with breast cancer were older (mean 58.5 vs. 55.4 years), higher proportion of White individuals (97.8 vs. 96.4%) and low-income (16.1 vs. 13.4%) and lower proportion of non-smoking (57.9 vs. 61.2%) and non-drinking (5.8 vs. 6.7%). Patients with breast cancer had higher HDPI (58.7 vs. 58.1) and AMED scores (4.4 vs. 4.3). Intake of vitamin A, B1, B9, B12, C, D, E, magnesium, iron, and copper were significantly different between the two groups (*P* < 0.05). For CLHLS, lower index groups were older (mean 83.9 vs. 81.0, 84.0 vs. 80.9; Supplementary Table S1). And there was significant difference between participants with higher index and participants with lower index in terms of province, financial support and years of schooling (*P* < 0.01).

**Table 1:** Baseline characteristics of participants in the UK Biobank.

**Characteristics**	**UK Biobank**		
**Breast cancer at baseline**	**No (*****N*** = **67,045)**	**Yes (*****N*** = **3,397)**	***p*** **-value**
**Demographics**			
Age, mean (SD), years	55.4 (7.7)	58.5 (6.8)	< 0.001
White, *n* (%)	64,648 (96.4)	3,322 (97.8)	< 0.001
Less than high school, *n* (%)	16,692 (24.9)	844 (24.9)	0.942
Low household income^*^, *n* (%)	9,008 (13.4)	546 (16.1)	< 0.001
Non-smoking, *n* (%)	40,995 (61.2)	1,966 (57.9)	< 0.001
Non-drinking, *n* (%)	4,495 (6.7)	196 (5.8)	0.015
History of diabetes, *n* (%)	2,062 (3.1)	89 (2.6)	0.132
BMI, mean (SD), kg/m^2^	26.3 (4.5)	26.2 (4.6)	0.508
Total energy intake, mean (SD), kcal	1,928.6 (582.1)	1,935.3 (564.6)	0.503
**Dietary patterns scores (SD)**			
AMED	4.3 (1.2)	4.4 (1.2)	< 0.001
HPDI	58.1 (6.4)	58.7 (6.2)	< 0.001
**Vitamins (SD)**			
Retinol (Vitamin A), mcg	427.6 (1,009.9)	470.8 (1,121.1)	0.028
Alpha-carotene, mcg	555.2 (796.1)	564.8 (772.7)	0.496
Beta-carotene, mcg	2,878.2 (3,026.2)	2,980.1 (2,965.8)	0.055
Thiamin (Vitamin B1), mg	1.8 (0.8)	1.8 (0.8)	0.014
Riboflavin (Vitamin B2), mg	1.8 (0.7)	1.9 (0.7)	0.276
Niacin (Vitamin B3), mg	35.9 (12.1)	35.9 (11.6)	0.656
Vitamin B6, mg	1.9 (0.7)	1.9 (0.7)	0.137
Folate (Vitamin B9), mcg	302.7 (114.2)	312.7 (117.0)	< 0.001
Vitamin B12, mcg	6.0 (3.7)	6.1 (3.9)	0.007
Vitamin C, mg	134.7 (88.0)	144.3 (89.9)	< 0.001
Vitamin D, mcg	3.4 (3.4)	3.6 (3.5)	< 0.001
Vitamin E, mg	10.9 (5.1)	11.2 (5.3)	< 0.001
**Minerals (SD)**			
Calcium, mg	955.3 (365.2)	950.8 (353.9)	0.484
Phosphorus, mg	1,375.8 (402.5)	1,379.8 (391.5)	0.571
Magnesium, mg	322 (98.1)	327.0 (98.2)	0.006
Iron, mg	11.8 (4.0)	12.0 (4.0)	0.002
Zinc, mg	9.3 (3.5)	9.3 (3.4)	0.871
Copper, mg	1.4 (0.6)	1.4 (0.6)	< 0.001
Sodium, mg	1,815.1 (808.2)	1,810.5 (786.4)	0.750
Selenium, mcg	51.0 (27.6)	51.9 (26.9)	0.073

Descriptive data were shown as mean (SD) while categorical variables were reported as n (%). ^*^In UK Biobank, household income < £18,000 was defined as low household income. *p*-value < 0.05 were considered significant. -, data not available. SD, standard deviation; N, number; BMI, body mass index; AMED, Alternate Mediterranean Diet; HPDI, Healthful Plant-Based Diet Index.

### 3.2 Associations of plant-based dietary patterns with incidence breast cancer and mortality

**Figure 1:**
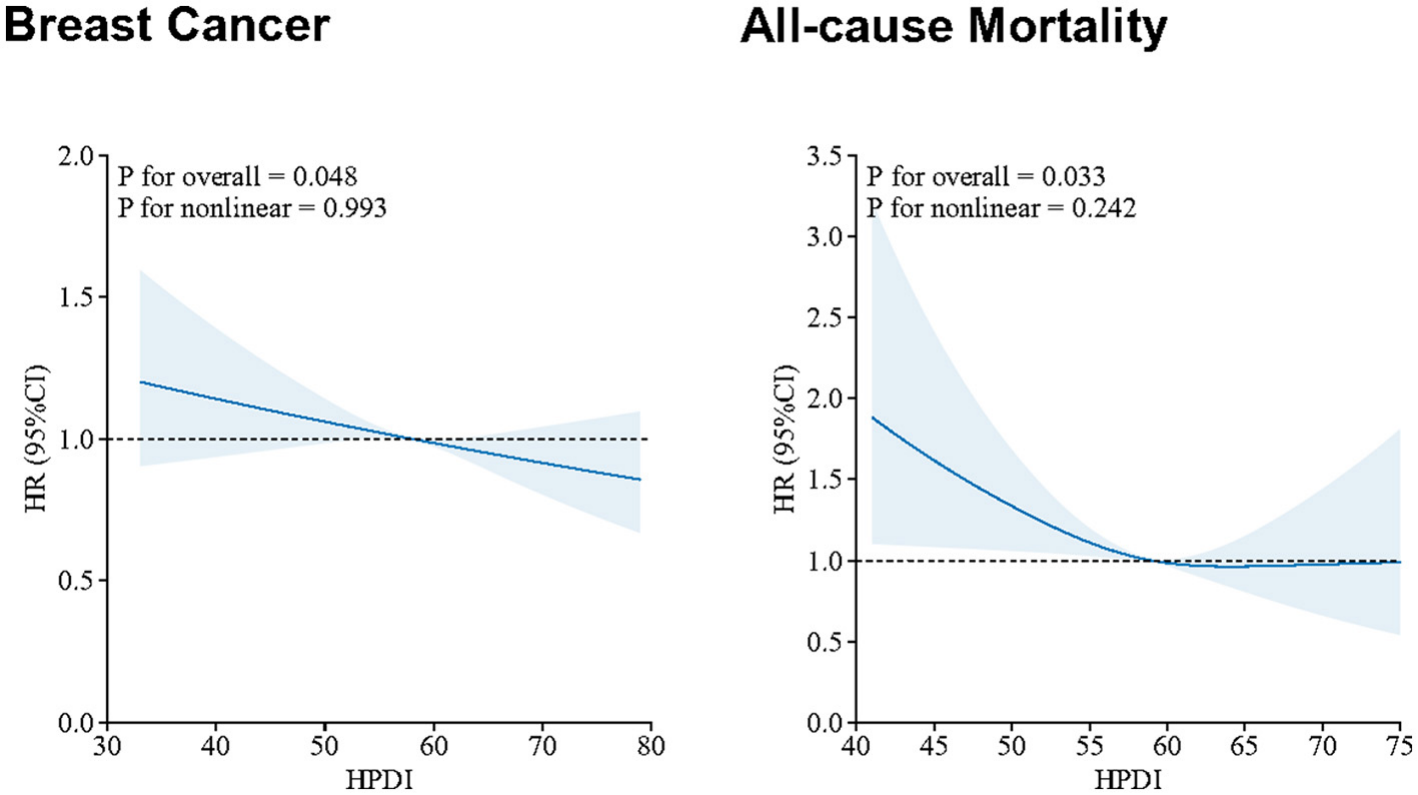
Association of plant-based diets and minerals with incidence breast cancer and mortality. Model 1: age (continuous), ethnicity/race (White, Asian or Asian British, Black or Black British, Chinese, Mixed, Other ethnic group), total energy intake (continuous). Model 2: Model 1+ BMI (continuous), educational level (less than high school, high school and above), Townsend deprivation index (T1, T2, T3), smoking status (Yes or No), drinking frequency (unknown, never, <1 time/week, 1–7 times/week). *P*-values less than 0.05 (*P* < 0.05) were considered significant. UKB, UK Biobank; BMI, body mass index; AMED, Alternate Mediterranean Diet; HPDI, Healthful Plant-Based Diet Index; HR, hazard ratio; CI, confidence interval; *N*, number.

**Figure 2:**
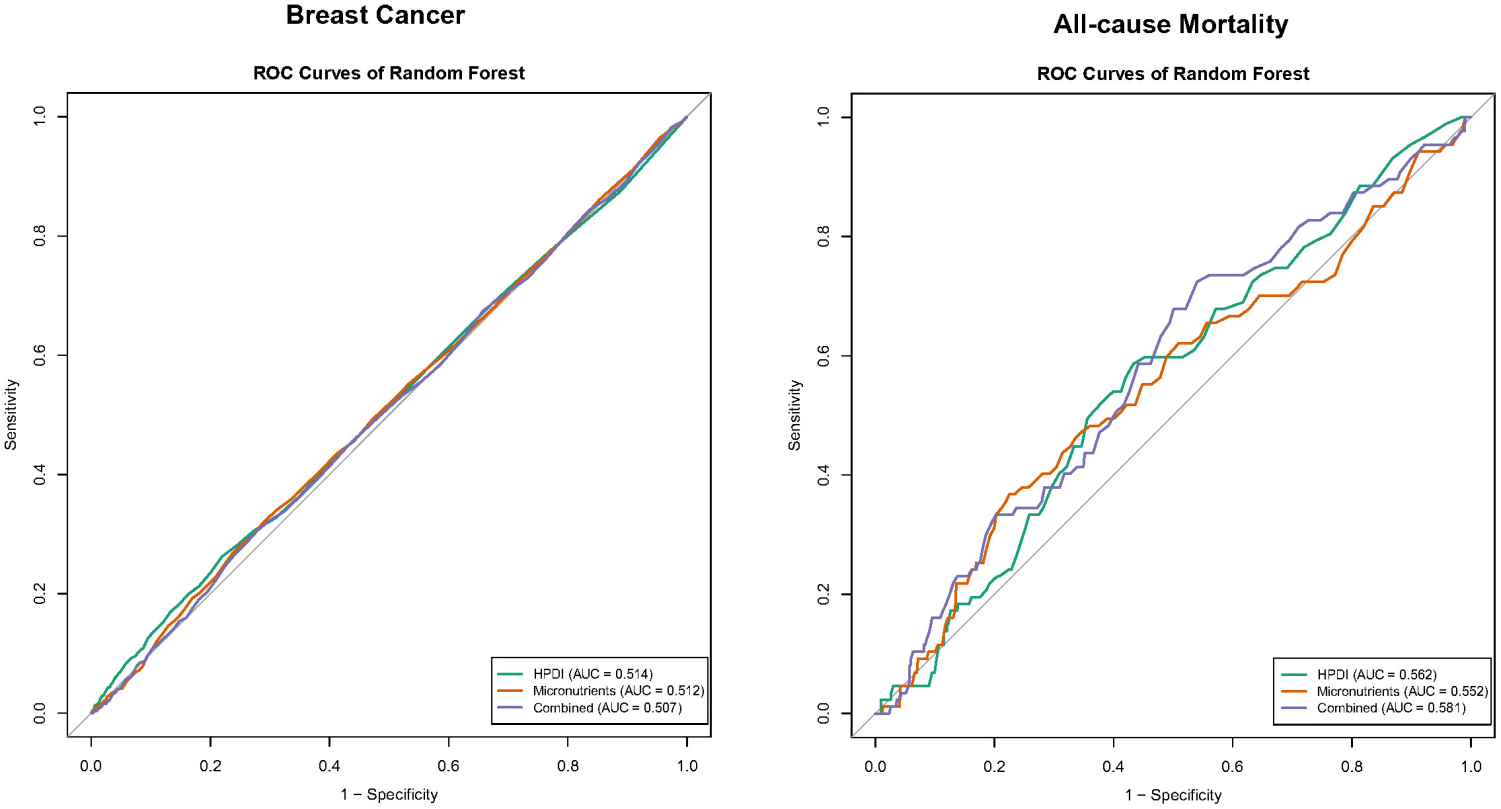
Association of plant-based diets with incidence cancer. Model 1: age (<90, ≥90), gender (male, female). Model 2: Model 1+ province (23 provinces and cities including Beijing, Tianjing, Hebei, and others), occupation (10 occupational statuses including professional and technical personnel and others), financial support (10 financial supports including retirement wages and others), educational level (classified by years of schooling: <6, 7–8, 9–11, ≥12), drinking status (yes, no), physical activity (yes, no), history of CVD (yes, no) and SBP (continuous). *P*-values less than 0.05 (*P* < 0.05) were considered significant. PDI, Plant-Based Diet Index; HPDI, Healthful Plant-Based Diet Index; HR, hazard ratio; CI, confidence interval; *N*, number.

**Figure 3:**
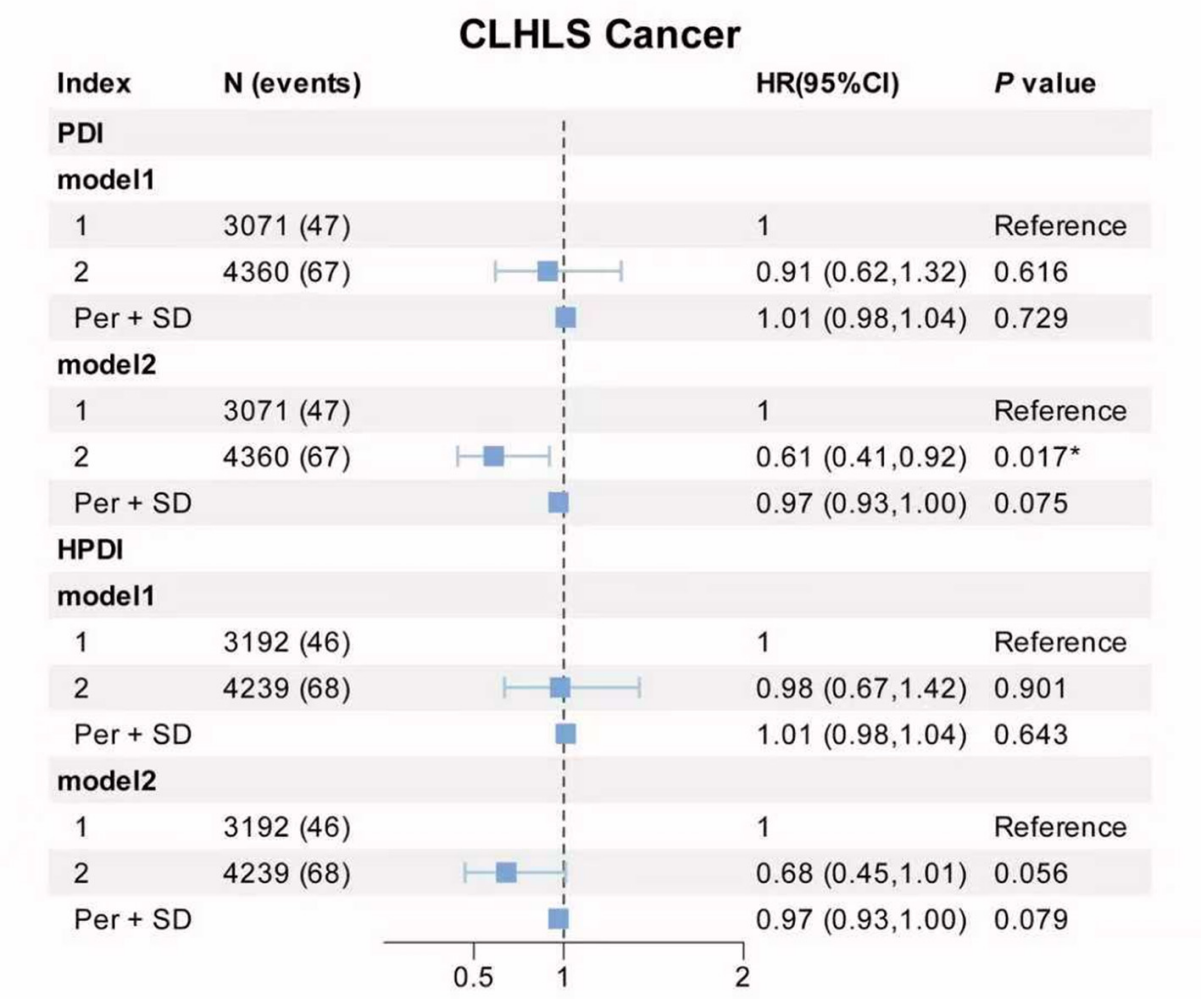
Restricted cubic spline plots of the association of HPDI with incidence breast cancer and mortality. Model 1: age (continuous), ethnicity/race (White, Asian or Asian British, Black or Black British, Chinese, Mixed, Other ethnic group), total energy intake (continuous). Model 2: Model 1+ BMI (continuous), educational level (less than high school, high school and above), Townsend deprivation index (T1, T2, T3), smoking status (Yes or No), drinking frequency (unknown, never, <1 time/week, 1–7 times/week). *P*-values less than 0.05 (*P* < 0.05) were considered significant. UKB, UK Biobank; BMI, body mass index; HPDI, Healthful Plant-Based Diet Index; HR, hazard ratio; CI, confidence interval; *N*, number.

As illustrated in Figure 1, HPDI exhibited significant associations with incidence breast cancer and all-cause mortality. Compared to the lowest tertile, the highest tertile of HPDI was associated with an 11% lower risk of new breast cancer [HR (95%CI): 0.89 (0.82, 0.98)]. Additionally, each SD increase in HPDI was associated with a 4% lower risk of new breast cancer [0.96 (0.93, 1.00)]. Similarly, among breast cancer patients, each SD increase in HPDI was associated with an 11% lower risk of all-cause mortality [0.89 (0.79, 1.00)], and the highest tertile of HPDI was associated with a 28% lower risk of all-cause mortality compared to the lowest tertile [0.72 (0.55, 0.95)]. The results of Model 1 and Model 2 were generally consistent. Higher AMED scores were associated with a decreased risk of new breast cancer and all-cause mortality, but were not statistically significant. In CLHLS, high PDI and HPDI are associated with a lower incidence rate of cancer (Figure 2). RCS analysis revealed no significant non-linear relationship between continuous HPDI and breast cancer or all-cause mortality (*P* for non-linear = 0.993 and 0.242, Figure 3). Compared with the low score group, the high score group of HPDI was associated with a 32% reduction in the risk of cancer in Model 2 [HR (95%CI): 0.68 (0.45, 1.01)], but the results were not statistically significant (*P* = 0.056). However, in Model 2 of the PDI index, the high score group was associated with a 39% reduction in the risk of cancer, and the results were statistically significant (*P* = 0.017).

### 3.3 Associations of micronutrients with incidence breast cancer and mortality

**Figure 4:**
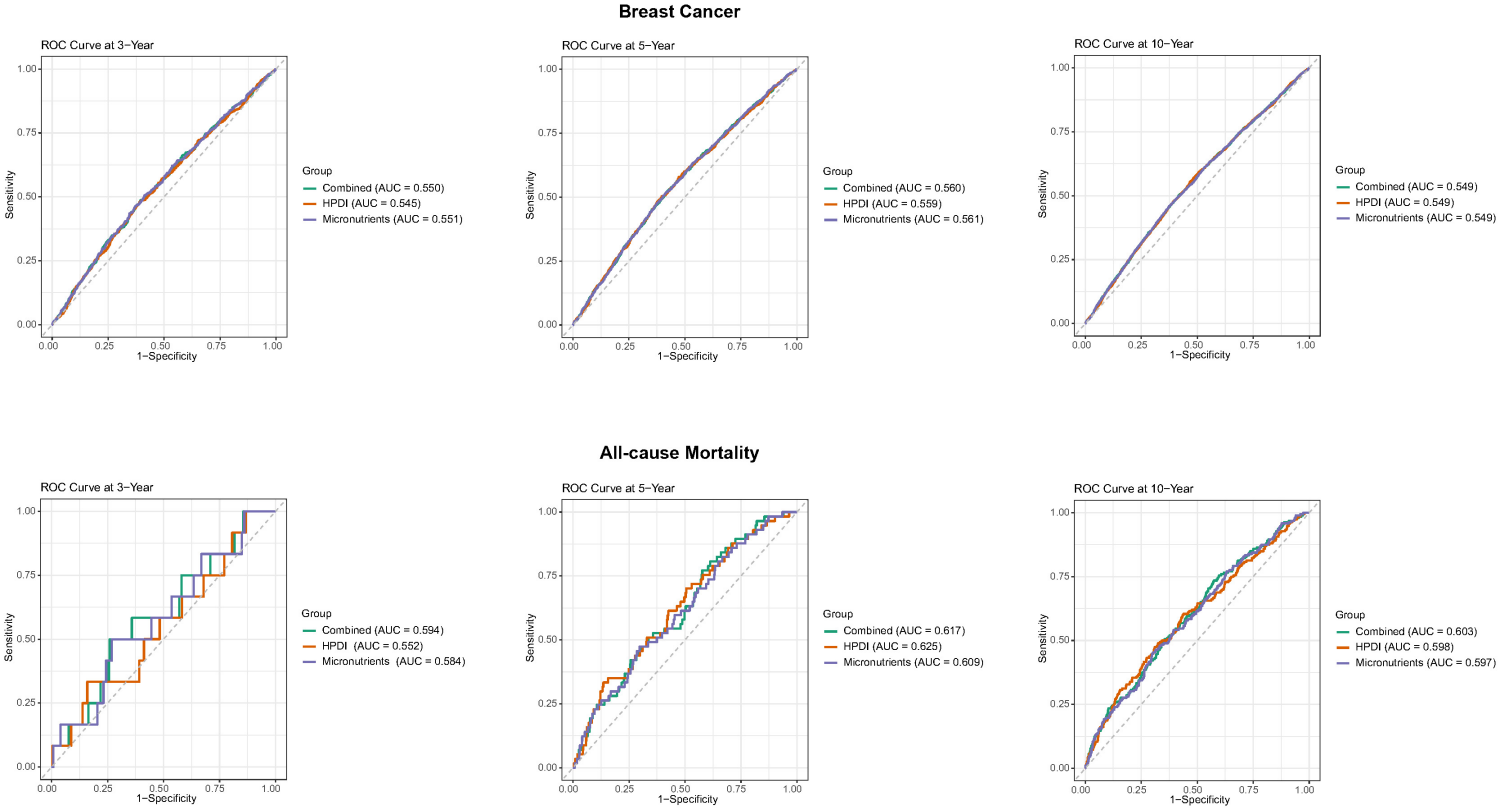
Association of vitamins with incidence breast cancer and mortality. Model 1: age (continuous), ethnicity/race (White, Asian or Asian British, Black or Black British, Chinese, Mixed, Other ethnic group), total energy intake (continuous). Model 2: Model 1+ BMI (continuous), educational level (less than high school, high school and above), Townsend deprivation index (T1, T2, T3), smoking status (Yes or No), drinking frequency (unknown, never, <1 time/week, 1–7 times/week). *P*-values less than 0.05 (*P* < 0.05) were considered significant. UKB, UK Biobank; BMI, body mass index; HR, hazard ratio; CI, confidence interval; *N*, number.

Figures 1, 4 illustrate the associations of mineral and vitamins intake with outcome. Compared to the lowest tertile, the highest tertile of calcium intake was associated with an 12% lower risk of new breast cancer [HR (95%CI): 0.88 (0.79, 0.98)]. Additionally, each SD increase in calcium intake was associated with a 5% lower risk of new breast cancer [0.95 (0.91, 1.00)]. Compared to the lowest tertile, the highest tertile of magnesium and copper intake was associated with an 11% [0.89 (0.79, 1.00)] and 12% [0.88 (0.79, 0.99)] lower risk of new breast cancer, respectively. Among breast cancer patients, each SD increase in calcium and phosphorus intake was associated with a 15% [0.85 (0.74, 0.98)] and 18% [0.82 (0.68, 0.98)] lower risk of all-cause mortality, respectively. In contrast, each SD increase in sodium intake was associated with a 15% increased risk of mortality [1.15 (1.01, 1.32)]. Furthermore, magnesium intake in the second tertile was associated with a 26% lower risk of all-cause mortality compared to the lowest tertile [0.74 (0.55, 0.99)].

Compared to the lowest tertile, the highest tertile of vitamin C intake was associated with a significant 9% lower risk of new breast cancer [0.91 (0.83, 0.99)]. Among breast cancer patients, the highest tertile of vitamin B2 intake, compared to the lowest tertile, was associated with a 27% lower risk of all-cause mortality [0.73 (0.53, 0.99)].

### 3.4 Random forest and time-dependent ROC curves and C-index of HPDI and micronutrients for predicting incidence breast cancer and all-cause mortality

Based on the results of the previous COX regression modeling, we selected micronutrients that were significantly associated with outcomes separately. In multivariate models adjusted for other clinically relevant variables, the C-index for new breast cancer was 0.5408 for HPDI and 0.5405 for micronutrients, respectively; however, the C-index increased to 0.5416 when HPDI and micronutrients were included jointly. For all-cause mortality among breast cancer patients, the C-index was 0.6041 for HPDI and 0.6104 for micronutrients, respectively, increasing to 0.6123 upon their combined inclusion (Supplementary Table S1).

**Figure 5:**
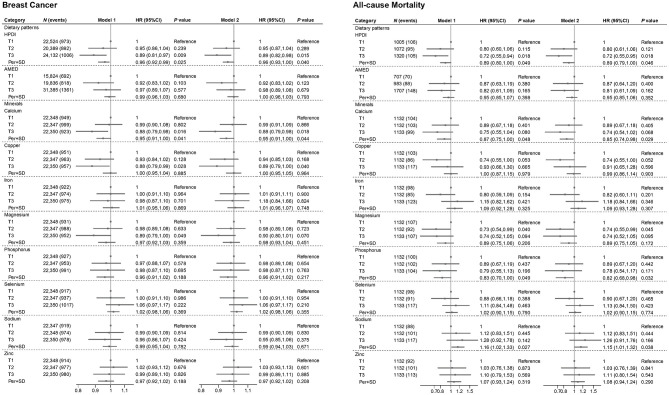
Random forest curves and random forest AUC values of HPDI and micronutrients for predicting incidence breast cancer and all-cause mortality. Micronutrients in breast cancer including vitamin C, calcium, copper and magnesium; Combined in breast cancer including HPDI, vitamin C, calcium, copper and magnesium; Micronutrients in all-cause mortality including vitamin B2, calcium, phosphorus, Sodium and magnesium; Combined in all-cause mortality including HPDI, vitamin B2, calcium, phosphorus, Sodium and magnesium; Model: age (continuous), ethnicity/race (UKB: White, Asian or Asian British, Black or Black British, Chinese, Mixed, Other ethnic group), total energy intake (continuous), educational level (less than high school, high school and above), Townsend deprivation index (T1, T2, T3), smoking status (Yes or No), drinking frequency (unknown, never, <1 time/week, 1–7 times/week), BMI (continuous). BMI, body mass index; HPDI, Healthful Plant-Based Diet Index; ROC, receiver operating characteristic; AUC, area under the ROC curve.

Figure 5 demonstrates the performance of HPDI, micronutrients, and their combination in predicting new breast cancer and all-cause mortality among breast cancer patients using the Random Forest model. For predicting new breast cancer, the AUC values were 0.514 for HPDI, 0.512 for micronutrients, and 0.507 for their combination. Among breast cancer patients, the AUC values for predicting all-cause mortality were 0.562 for HPDI, 0.552 for micronutrients, and 0.581 for the combined model.

**Figure 6:**
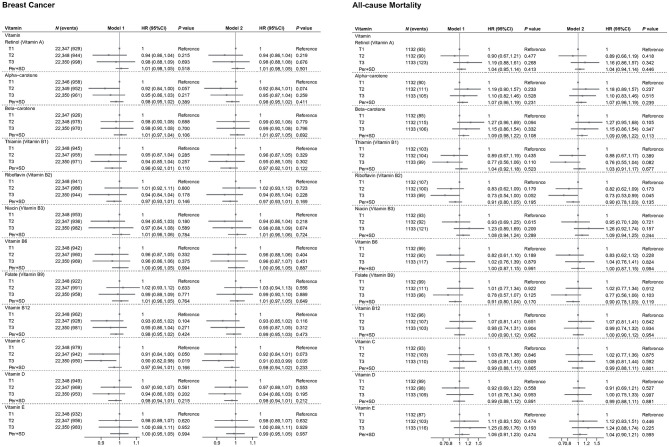
Time-dependent ROC curves and time-dependent AUC values of HPDI and micronutrients for predicting incidence breast cancer and all-cause mortality. Micronutrients in breast cancer including vitamin C, calcium, copper and magnesium; Combined in breast cancer including HPDI, vitamin C, calcium, copper and magnesium; Micronutrients in all-cause mortality including vitamin B2, calcium, phosphorus, Sodium and magnesium; Combined in all-cause mortality including HPDI, vitamin B2, calcium, phosphorus, Sodium and magnesium; Model: age (continuous), ethnicity/race (UKB: White, Asian or Asian British, Black or Black British, Chinese, Mixed, Other ethnic group), total energy intake (continuous), educational level (less than high school, high school and above), Townsend deprivation index (T1, T2, T3), smoking status (Yes or No), drinking frequency (unknown, never, <1 time/week, 1–7 times/week), BMI (continuous). BMI, body mass index; HPDI, Healthful Plant-Based Diet Index; ROC, receiver operating characteristic; AUC, area under the ROC curve.

We developed time-dependent ROC modeling to evaluate the performance of HPDI, micronutrients individually, and their combination in predicting new breast cancer and mortality at 3, 5, and 10 years. The results indicated that predictive performance for both outcomes was generally optimal at the 5-year mark for all predictors (HPDI alone, micronutrients alone, and their combination). Specifically, micronutrient intake alone demonstrated the highest AUC for predicting new breast cancer across all three time points (3, 5, and 10 years). In contrast, HPDI alone achieved its best performance for predicting mortality among breast cancer patients at 5 years, with an AUC of 0.625 (Figure 6). Notably, the combination of HPDI and micronutrients yielded the highest AUC for predicting both new breast cancer and mortality at the 10-year time point.

## 4 Discussion

In this large prospective cohort of UKB participants, greater adherence to a HPDI was significantly associated with reduced breast cancer incident and improved overall survival among breast cancer patients. Specifically, women in the highest HPDI tertile experienced an 11% lower risk of developing breast cancer and a 28% lower risk of all-cause mortality after diagnosis, while selected micronutrients (including calcium, magnesium, copper, phosphorus, vitamin C, and vitamin B_2_) showed independent inverse associations with these outcomes, whereas sodium intake was positively associated with mortality. Although the predictive performance of HPDI, micronutrients, and their combination was modest, the incremental improvement observed when combining dietary pattern and nutrient data suggests that comprehensive dietary profiling may offer additional, albeit limited, predictive value for long-term breast cancer outcomes.

Our findings are consistent with prior cohort studies reporting plant-forward diets or dietary quality indices (e.g., HPDI, PDI) to reduced breast cancer risk. Alignment with limited evidence linking post-diagnosis dietary quality to survival benefits. Meta-analyses have shown 10%−15% reductions in incidence among women adhering to healthful plant-forward diets or vegetable-fruit-soybean patterns (18). Our observed 11% lower risk in the highest HPDI tertile aligns closely with these estimates. Similarly, our finding of a 28% lower mortality risk aligns with prior evidence suggesting improved survival among patients with higher dietary quality after diagnosis (19). We observed that the non-linear association between the HPDI and breast cancer incidence as well as all-cause mortality was not statistically significant, with a tendency toward a negative correlation. The non-significant trend observed for AMED may reflect the index's lower sensitivity in distinguishing plant- vs. animal-derived food quality within the UK dietary context. As prior work noted, dietary variables alone provide modest discriminatory power compared with molecular or genetic models (e.g., Molecular Classification of Breast Cancer), our predictive performance metrics (C-index and AUC) remained limited even when combining HPDI and micronutrients, reinforcing the need to integrate diet with other established risk factors for meaningful clinical prediction. In the analysis of individual nutrient effects, we found that vitamin C intake in the highest tertile was associated with a 9% lower risk of incident breast cancer compared with the lowest tertile, a relationship plausibly attributable to vitamin C's robust antioxidant properties. Meanwhile, vitamin B2 was shown to exert a significant protective effect on mortality risk among breast cancer patients, thereby providing a theoretical rationale for nutritional intervention strategies targeting this population.

A plant-based dietary pattern demonstrates favorable preventive effects and improved prognosis in female breast cancer. Consistent with this trend, our CLHLS study similarly found that higher PDI and HPDI levels were associated with a 39 and 32% reduction in overall cancer risk, respectively. Although data limitations prevented separate analysis of breast cancer cases, the findings still indicate that HPDI and PDI are linked to reduced overall cancer risk, providing supplementary evidence for the cancer-preventive potential of plant-based diets.

Our study benefits from the exceptionally large sample size and rich phenotypic data of the UKB, enabling robust estimates and simultaneous evaluation of overall dietary patterns and individual micronutrients. The prospective design, use of multivariable Cox models, restricted cubic splines, and machine learning approaches further strengthen the validity of our observations. However, the observational nature of the analysis precludes causal inference and residual confounding cannot be fully excluded. Due to inherent limitations in the availability of UK Biobank data, the database lacks systematic questionnaire data on Hormone replacement therapy, family history of breast cancer, and parity. Menopausal status could only be inferred from estradiol levels, which were available for only a subset of participants. Consequently, these breast cancer risk factors were not included in the model. Dietary intake was assessed only once at baseline, potentially underestimating temporal changes. Predictive performance remained modest despite combining HPDI and micronutrients; and the predominance of White participants limits generalizability to more diverse populations. Additionally, the constraints of self-reported data in the CLHLS preclude the specific identification of breast cancer, limiting our capacity to evaluate the distinct impacts of HPDI and PDI on breast cancer-related outcomes in the Chinese cohort.

In summary, greater adherence to a healthful plant-based diet and optimal intake of selected micronutrients were associated with lower breast cancer incidence and improved survival, although predictive performance was limited. These findings underscore the potential public health importance of promoting plant-forward dietary patterns and adequate micronutrient intake as part of comprehensive breast cancer prevention and survivorship strategies. Future prospective and interventional studies across diverse populations are warranted to clarify causality and refine dietary risk prediction models.

## 5 Conclusions

High adherence to healthful plant-based diets (e.g., HPDI/PDI) combined with adequate intake of key micronutrients like calcium and magnesium improves breast health, reduces breast cancer incidence, and enhances long-term survival in patients.

## Data Availability

The raw data supporting the conclusions of this article will be made available by the authors, without undue reservation.
