# Supplementary material for: Plant-based dietary patterns, micronutrient status and breast cancer outcomes: a joint analysis of UK Biobank and Chinese longitudinal healthy longevity survey
Source: Front Nutr. 2026 Jan 26;12:1748611. doi: 10.3389/fnut.2025.1748611 (PMC12883384; doi:10.3389/fnut.2025.1748611)
Supplement: Supplementary file 1 [file Table_1.docx]

**Table S1.** Baseline characteristics of participants in the CLHLS.

| **CLHLS** | | | | | | |
| --- | --- | --- | --- | --- | --- | --- |
| **Index level** | **Lower PDI**  **(N = 3,071)** | **Higher PDI**  **(N = 4,360)** | **P value** | **Lower HPDI**  **(N =3,192)** | **Higher HPDI**  **(N = 4,239)** | **P value** |
| Age, mean (SD), years | 83.9 (11.0) | 81.0 (10.7) | < 0.001 | 84.0 (10.9) | 80.9 (10.8) | < 0.001 |
| Male, n(%) | 1314 (42.8) | 2068 (47.4) | < 0.001 | 1348 (42.2) | 2034 (48.0) | < 0.001 |
| Province, n(%) |  |  | < 0.001 |  |  | < 0.001 |
| Beijing | 32 (1.0) | 117 (2.7) |  | 56 (1.8) | 93 (2.2) |  |
| Tianjing | 13 (0.4) | 32 (0.7) |  | 20 (0.6) | 25 (0.6) |  |
| Hebei | 14 (0.5) | 48 (1.1) |  | 26 (0.8) | 36 (0.8) |  |
| Shanxi | 21 (0.7) | 50 (1.1) |  | 22 (0.7) | 49 (1.2) |  |
| Liaoning | 114 (3.7) | 164 (3.8) |  | 99 (3.1) | 179 (4.2) |  |
| Jilin | 34 (1.1) | 123 (2.8) |  | 53 (1.7) | 104 (2.5) |  |
| Helongjiang | 61 (2.0) | 57 (1.3) |  | 42 (1.3) | 76 (1.8) |  |
| Shanghai | 72 (2.3) | 71 (1.6) |  | 80 (2.5) | 63 (1.5) |  |
| Jiangsu | 159 (5.2) | 475 (10.9) |  | 220 (6.9) | 414 (9.8) |  |
| Zhejiang | 84 (2.7) | 482 (11.1) |  | 105 (3.3) | 461 (10.9) |  |
| Anhui | 123 (4.0) | 209 (4.8) |  | 131 (4.1) | 201 (4.7) |  |
| Fujian | 27 (0.9) | 51 (1.2) |  | 25 (0.8) | 53 (1.3) |  |
| Jiangxi | 54 (1.8) | 97 (2.2) |  | 27 (0.8) | 124 (2.9) |  |
| Shangdong | 325 (10.6) | 624 (14.3) |  | 378 (11.8) | 571 (13.5) |  |
| Henan | 186 (6.1) | 325 (7.5) |  | 181 (5.7) | 330 (7.8) |  |
| Hubei | 48 (1.6) | 245 (5.6) |  | 62 (1.9) | 231 (5.4) |  |
| Hunan | 132 (4.3) | 229 (5.3) |  | 135 (4.2) | 226 (5.3) |  |
| Guangdong | 217 (7.1) | 136 (3.1) |  | 174 (5.5) | 179 (4.2) |  |
| Guangxi | 749 (24.4) | 249 (5.7) |  | 638 (20.0) | 360 (8.5) |  |
| Hainan | 150 (4.9) | 20 (0.5) |  | 154 (4.8) | 16 (0.4) |  |
| Chongqing | 165 (5.4) | 107 (2.5) |  | 181 (5.7) | 91 (2.1) |  |
| Sichuan | 262 (8.5) | 374 (8.6) |  | 360 (11.3) | 276 (6.5) |  |
| Shaanxi | 29 (0.9) | 75 (1.7) |  | 23 (0.7) | 81 (1.9) |  |
| Occupation, n(%) |  |  | 0.059 |  |  | < 0.001 |
| professional and technical personnel | 129 (4.2) | 207 (4.7) |  | 106 (3.3) | 230 (5.4) |  |
| governmental, institutional or managerial personnel | 85 (2.8) | 181 (4.2) |  | 71 (2.2) | 195 (4.6) |  |
| commercial, service or industrial worker | 352 (11.5) | 602 (13.8) |  | 355 (11.1) | 599 (14.1) |  |
| self-employed | 57 (1.9) | 78 (1.8) |  | 50 (1.6) | 85 (2.0) |  |
| agriculture, forestry, animal husbandry or fishery worker | 2173 (70.8) | 2882 (66.1) |  | 2315 (72.5) | 2740 (64.6) |  |
| houseworker | 186 (6.1) | 269 (6.2) |  | 200 (6.3) | 255 (6.0) |  |
| military personnel | 20 (0.7) | 35 (0.8) |  | 24 (0.8) | 31 (0.7) |  |
| never worked | 18 (0.6) | 23 (0.5) |  | 15 (0.5) | 26 (0.6) |  |
| others | 51 (1.7) | 83 (1.9) |  | 56 (1.8) | 78 (1.8) |  |
| Financial support, n(%) |  |  | < 0.001 |  |  | < 0.001 |
| retirement wages | 513 (16.7) | 901 (20.7) |  | 481 (15.1) | 933 (22.0) |  |
| spouse | 83 (2.7) | 139 (3.2) |  | 82 (2.6) | 140 (3.3) |  |
| child(ren) | 1777 (57.9) | 2305 (52.9) |  | 1882 (59.0) | 2200 (51.9) |  |
| grandchild(ren) | 113 (3.7) | 74 (1.7) |  | 98 (3.1) | 89 (2.1) |  |
| other relative(s) | 13 (0.4) | 17 (0.4) |  | 14 (0.4) | 16 (0.4) |  |
| local government or community | 183 (6.0) | 206 (4.7) |  | 196 (6.1) | 193 (4.6) |  |
| work by self | 342 (11.1) | 662 (15.2) |  | 389 (12.2) | 615 (14.5) |  |
| others | 47 (1.5) | 56 (1.3) |  | 50 (1.6) | 53 (1.3) |  |
| Years of schooling, mean (SD), years | 2.21 (3.48) | 2.58 (3.68) | < 0.001 | 1.99 (3.19) | 2.75 (3.86) | < 0.001 |
| Non-drinking, n(%) | 2504 (81.5) | 3458 (79.3) | 0.060 | 2588 (81.1) | 3374 (79.6) | 0.283 |
| Physical activity, n(%) | 954 (31.1) | 1528 (35.0) | 0.002 | 1040 (32.6) | 1442 (34.0) | 0.430 |
| History of CVD, n(%) | 408 (13.3) | 597 (13.7) | 0.880 | 402 (12.6) | 603 (14.2) | 0.126 |
| SBP, mean (SD), mmHg | 137 (21.2) | 138 (21.6) | 0.221 | 137 (21.3) | 138 (21.6) | 0.824 |

**Note:** Descriptive data were shown as mean (SD) while categorical variables were reported as n (%). P value < 0.05 were considered significant. - :data not available.

**Abbreviations:** SD=standard deviation, N=number, PDI=Plant-Based Diet Index, HPDI=Healthful Plant-Based Diet Index.
