# Supplementary material for: Plant-based dietary patterns, micronutrient status and breast cancer outcomes: a joint analysis of UK Biobank and Chinese longitudinal healthy longevity survey
Source: Front Nutr. 2026 Jan 26;12:1748611. doi: 10.3389/fnut.2025.1748611 (PMC12883384; doi:10.3389/fnut.2025.1748611)
Supplement: Supplementary file 2 [file Table_2.docx]

**Table S2.** C-index of the association of HPDI and micronutrients with new breast cancer and mortality based on COX regression model.

| **Outcomes** | **C-index** |
| --- | --- |
| **Breast cancer** |  |
| HPDI | 0.5407842 |
| Micronutrients^a^ | 0.5405082 |
| Combined^b^ | 0.5416375 |
| **All-cause mortality** |  |
| HPDI | 0.6041448 |
| Micronutrients^c^ | 0.6103647 |
| Combined^d^ | 0.6122985 |

**Note:**

^a^Micronutrients including vitamin C, calcium, copper and magnesium;

^b^Combined including HPDI, vitamin C, calcium, copper and magnesium;

^c^Micronutrients including vitamin B2, calcium, phosphorus, Sodium and magnesium;

^d^ Combined including HPDI, vitamin B2, calcium, phosphorus, Sodium and magnesium;

Model adjusted for age (continuous), ethnicity/race (UKB: White, Asian or Asian British, Black or Black British, Chinese, Mixed, Other ethnic group), total energy intake (continuous), educational level (less than high school, high school and above), Townsend deprivation index (T1, T2, T3), smoking status (Yes or No), drinking frequency (unknown, never, <1 time/week, 1-7 times/week), BMI (continuous).

**Abbreviations:** BMI=body mass index, HPDI=Healthful Plant-Based Diet Index.
