# Supplementary figures and images for: Plant-based dietary patterns, micronutrient status and breast cancer outcomes: a joint analysis of UK Biobank and Chinese longitudinal healthy longevity survey
Source: Front Nutr. 2026 Jan 26;12:1748611. doi: 10.3389/fnut.2025.1748611 (PMC12883384; doi:10.3389/fnut.2025.1748611)

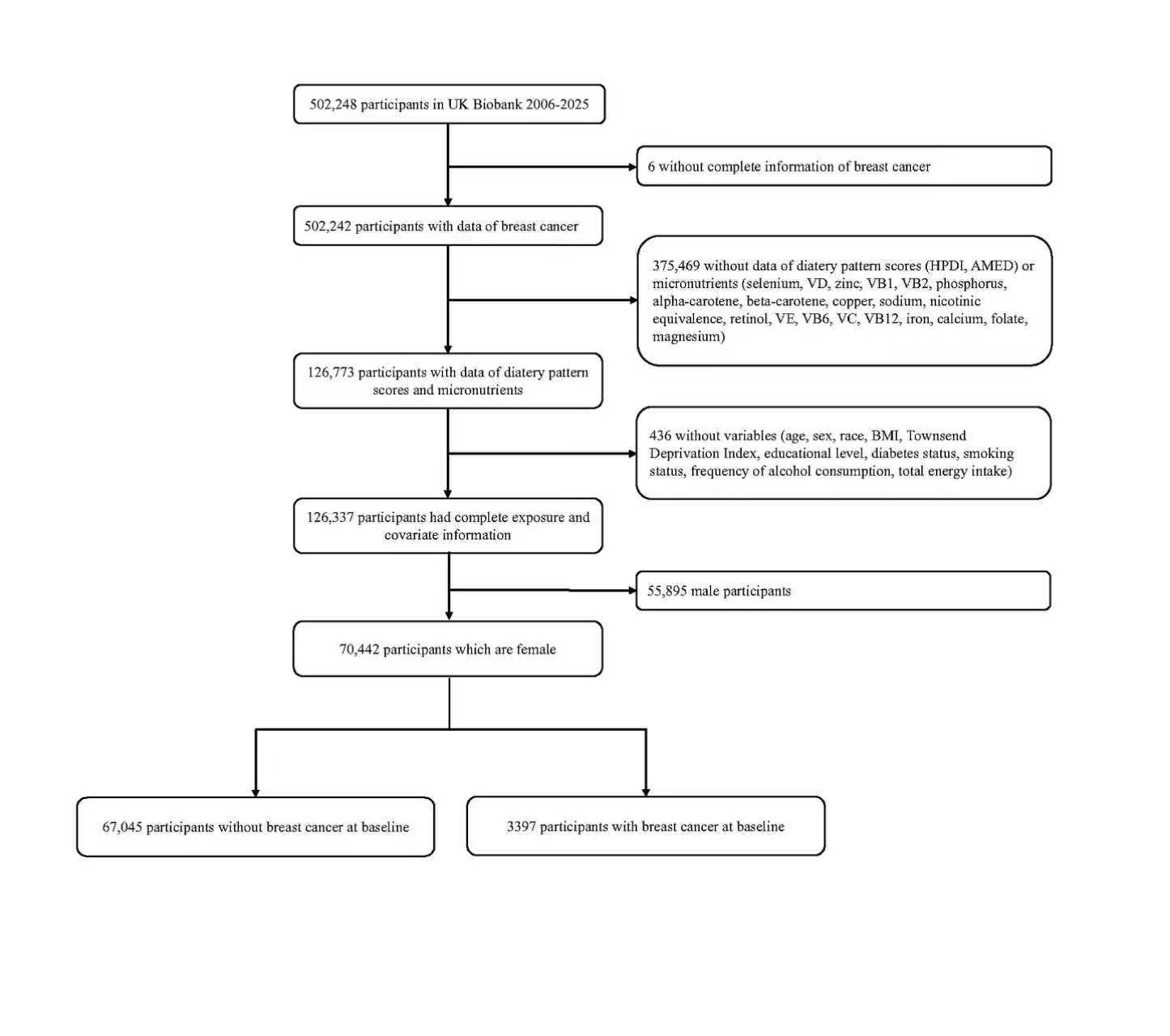

Supplement: Supplementary file 3 [file Image_1.JPEG]

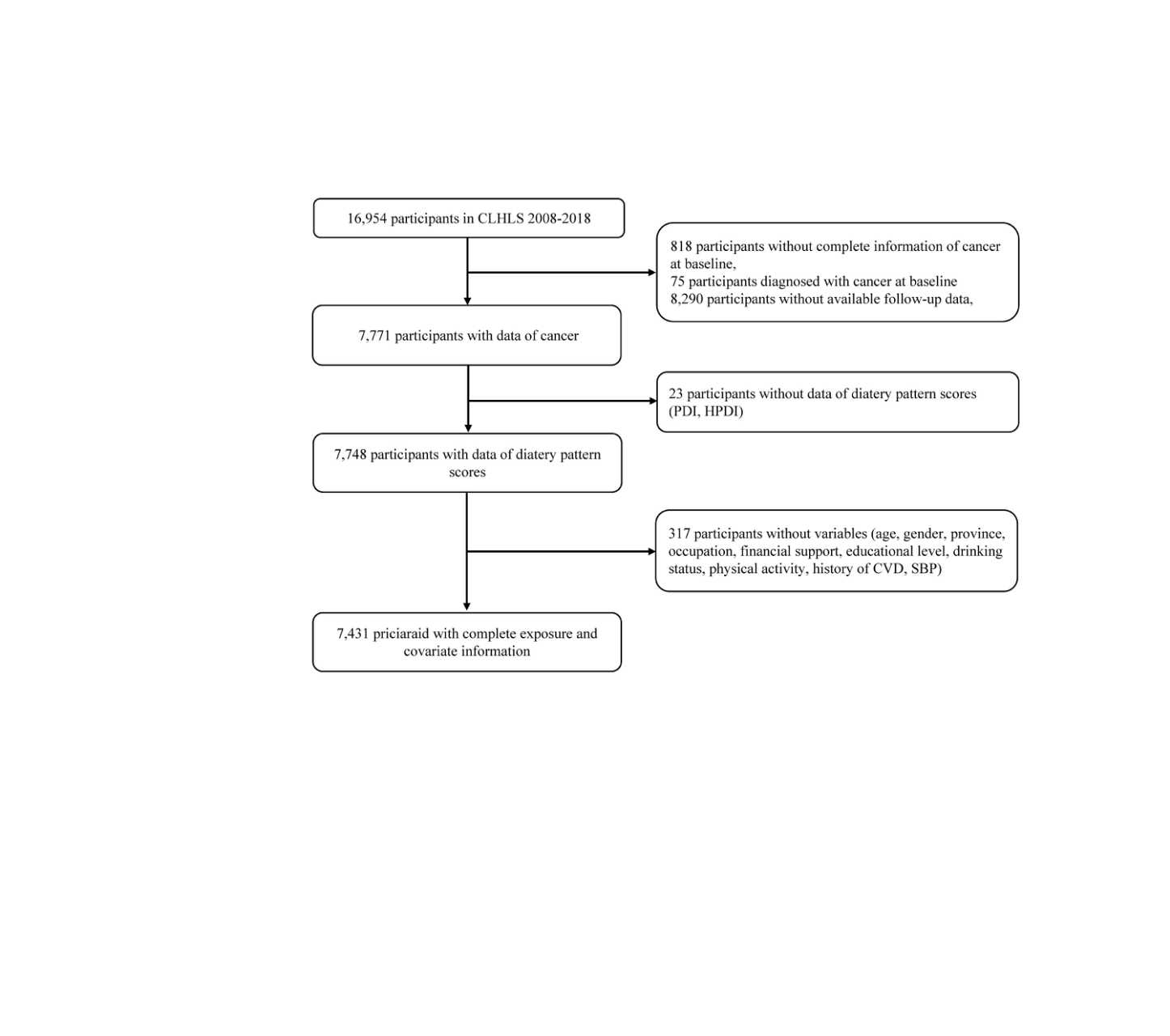

Supplement: Supplementary file 4 [file Image_2.JPEG]
